# On species delimitation, hybridization and population structure of cassava whitefly in Africa

**DOI:** 10.1038/s41598-021-87107-z

**Published:** 2021-04-12

**Authors:** S. Elfekih, W. T. Tay, A. Polaszek, K. H. J. Gordon, D. Kunz, S. Macfadyen, T. K. Walsh, S. Vyskočilová, J. Colvin, P. J. De Barro

**Affiliations:** 1 Australian Centre for Disease Preparedness, CSIRO, Geelong, VIC Australia; 2 Black Mountain Laboratories, CSIRO, Canberra, ACT Australia; 3 Department of Life Sciences, Natural History Museum, London, UK; 4 The Gurdon Institute, University of Cambridge, Tennis Court Road, Cambridge, CB2 1QN UK; 5 Natural Resources Institute, University of Greenwich, Kent, UK; 6 CSIRO, Ecosciences Precinct, Brisbane, Australia

**Keywords:** Ecological genetics, Evolutionary ecology, Invasive species, Molecular ecology, Population genetics

## Abstract

The *Bemisia* cassava whitefly complex includes species that cause severe crop damage through vectoring cassava viruses in eastern Africa. Currently, this whitefly complex is divided into species and subgroups (SG) based on very limited molecular markers that do not allow clear definition of species and population structure. Based on 14,358 genome-wide SNPs from 62 *Bemisia* cassava whitefly individuals belonging to sub-Saharan African species (SSA1, SSA2 and SSA4), and using a well-curated mtCOI gene database, we show clear incongruities in previous taxonomic approaches underpinned by effects from pseudogenes. We show that the SSA4 species is nested within SSA2, and that populations of the SSA1 species comprise well-defined south-eastern (Madagascar, Tanzania) and north-western (Nigeria, Democratic Republic of Congo, Burundi) putative sub-species. Signatures of allopatric incipient speciation, and the presence of a ‘hybrid zone’ separating the two putative sub-species were also detected. These findings provide insights into the evolution and molecular ecology of a highly cryptic hemipteran insect complex in African, and allow the systematic use of genomic data to be incorporated in the development of management strategies for this cassava pest.

## Introduction

Confidence in species identification provides insights into the evolutionary forces that drive species diversification, and it underpins the strategies for biological conservation and the management of natural resources. While morphological differences have enabled many species to be identified, cryptic species complexes present a particular challenge due to their general lack of readily distinguishable morphological characters^1–3^. Ecological and life history data including host plant use and mating behaviour are needed to mitigate and address these challenges^3–5^. This is often time-consuming and impractical especially with large numbers of samples across wide geographic range. Incorporating DNA markers, e.g., diagnostic mitochondrial DNA (mtDNA) markers^6–8^, genome-wide sequencing^9,10^, and whole-genome resequencing^11^ can increase confidence in differentiating cryptic species in a complex and identifying interspecific hybrids.

The challenges can become critical when individual cryptic species in a complex have a serious impact on plant health or agricultural productivity: insect vectors of plant viral diseases affecting cassava (*Manihot esculenta*), the most important food crop grown in Africa^12^, is one example of such case. Cassava production is vulnerable to cassava mosaic disease (CMD) caused by a number of cassava mosaic geminiviruses (CMGs)^13,14^ and CMD has been reported in Africa since the early 1970s^15^ and more recently in South-East Asia^16,17^. While the vector responsible for the spread of the CMD in South-East Asia is not well-understood, the pandemic in east and central Africa was associated with the distribution of sub-Saharan African (SSA) species of the *Bemisia* whitefly complex^13^. Invasive whitefly species such as the Mediterranean (MED), Middle East-Asia Minor 1 (MEAM1), and SSA2, have become pests of global agriculture, spreading plant viral diseases and becoming resistant to chemical control agents^18,19^. In particular, the endemic African cassava *Bemisia* whitefly SSA2 species occupies regions affected by the CMD pandemic. SSSA2 has also expanded into southern Europe since 2007^20^ whereas SSA1 has not (though widespread throughout the African continent). Defining the actual CMD vectors in Africa remains a challenge due to the cryptic nature of the many species in the Aleyrodidae whitefly genus *Bemisia*^3,18,21,22^.

Historically, the lack of distinguishable morphological characters among *Bemisia tabaci* has resulted in geographically distinct species being reclassified as a single species^23–25^ and underpinned the *B. tabaci* taxonomic confusion (e.g., review by De Barro et al.^18^; see also Tay et al.^26,27^). Currently, over 38 cryptic species are recognised within the *B. tabaci* (Gennadius) species complex^24^ through molecular diagnostics of a partial (i.e., 657 bp) mitochondrial DNA cytochrome oxidase subunit I (mtCOI)^18,28,29^, mating experiments^5,30,31^ (although low numbers of F1 hybrids^10,32^ including between SSA1 and SSA2 (previously ‘Ug1’ and ‘Ug2’)^33,34^ are known), behavioural^4^, and host preference^3^ studies.

Re-analyses of partial mtCOI sequences relating to the MED species^3^, and of the *B. tabaci* standard partial mtCOI dataset^33^ demonstrated that *ca*. 60–65% were nuclear mitochondrial DNA sequences (NUMTs) or other PCR artefacts^10,29,35^. These analyses, together with molecular characterisation of original voucher specimens, have begun to disentangle the species complex^26,27^, identified historical species ranges^26,27,36^, and provided phylogenetic evidence that the ‘*B. tabaci* cryptic species complex’ is a polyphyletic group derived from various SSA *Bemisia* whitefly species’^29^. Delimitation of populations across the full African *Bemisia* species habitat range will be needed. This would help to ascertain whether any of these species, e.g., SSA1, is genetically distinct with limited gene flow from others either due to external (e.g., geographic or climatic limitation) or internal (e.g., presence/absence of secondary symbionts) factors including host association.. Recognition of such taxonomic classifications and their distribution in sub-Saharan Africa will be important for management of these pest species that could pose a threat to plant health and global agricultural biosecurity^37^.

The advent of high-throughput sequencing (HTS) technologies has driven the availability of genome-wide data that is further transforming the *Bemisia* taxonomy. HTS has demonstrated that various species previously recognised via characterisation of mtCOI (e.g., SSA4^29^; Middle East Asia Minor 2 (MEAM2)^10,29,35^) were likely artefacts caused by the sub-optimal primers^16,17^ and sequencing of NUMTs^10,35^. Analyses of mitochondrial genomes also demonstrated inconsistencies of *B. tabaci* cryptic species identification based on the standard partial mtCOI gene region, and cautioned the need to combine behavioural and molecular evidence to delimitate species status^3,31^.

Despite the recent advances and their importance in the African agricultural landscape, the taxonomy, population structure, and evolutionary genetics of the African cassava Bemisia whitefly species complex (i.e., SSA1, SSA2, SSA3, SSA6, and SSA9)^29^ remain poorly understood. A recent study of taxonomic structure and gene flow among genetically diverse SSA1, SSA2, SSA3, and SSA4 species^38^ described serious discrepancies between their results obtained from mtCOI and from nextRAD sequencing. This (the conflicting results of mtCOI and nextRAD sequencing) findings has strongly implies that the species delimitation of sub-Sahara African *Bemisia* species needs to be revisited since it is currently based on mtCOI. We seek to show why mtCO and nuclear DNA should result in such conflicting implications for the African cassava *Bemisia* whitefly SSA1 (which is also further refined into subgroups (SG) 1, 2, and 3 in, e.g., Legg et al.^13^), SSA2, and SSA4 species. With the cassava *Bemisia* whitefly being the most common whitefly on cassava crops in Africa, and capable of vectoring diseases (e.g., cassava mosaic begamoviruses, CMBs; cassava brown streak ipomoviruses, CBSIs) known to cause significant crop losses^39^, our findings will also provide insights into incipient speciation of this agriculturally important *Bemisia* species complex with the capacity to impact on food security in sub-Saharan Africa^40,41^.

## Results

Wosula et al.^38^ reported a total of 25 partial mtCOI sequences from their 72 SSA samples studied. Of these 25 sequences, only 11 represented unique (non-redundant) mtCOI haplotypes [seven SSA1: MF417579, MF417581, MF417585, MF417587, MF417590, MF417592, MF417601; one SSA3: MF417591; one SSA2: MF417597; two ‘putative SSA4’ MF417589, MF417593 (with *ca*. 94% nucleotide identity to SSA3)].

### Integrity of African cassava *Bemisia* SSA mtCOI sequences

Berry et al.^42^ reported 11 ‘Sub-Saharan III: Western Africa-Cameroon/Cassava’ sequences which were renamed as SSA4^18^. Of these 11 SSA4 sequences, only two short sequences (i.e., Cam Ayos 1 WO2, Cam Ayos 2 WO3) did not have INDELs or exhibit any premature stop codons at the amino acid level (Suppl. Fig. 1). The remaining nine sequences had random INDELs that resulted in frame-shift mutations and premature stop codons, which suggested that they are likely to be NUMTs. The Cam Ayos 2 WO3 sequence also had nucleotide substitutions that resulted in amino acid residue changes in highly conserved mtCOI regions, suggesting that this was likely also a NUMT sequence. The remaining single, short but ‘functional’ Cam Ayos 1 WO2 sequence (AF344246) that phylogenetically grouped^42^ with the 10 NUMT-related SSA4 sequences suggested that as a whole, these SSA4 sequences originally reported by Berry et al.^42^ were all likely NUMTs. The fact that SSA4 sequences were phylog enetically clustered to SSA2 suggested they were also potentially of nuclear origin, similar to that reported for *B. tabaci* MEAM2 NUMT^10,35^ that originated from the nuclear genome of MEAM1^10,35^.

Further evidence that the partial mtCOI gene lacks the power to differentiate between evolutionary very closely related cryptic species (e.g., the ‘subgroups’ (SG)^13,43^ taxonomic status within SSA1) can be seen by examining nucleotide substitution patterns. For example, re-assessment of the two SSA1-SG5 sequences (MF417585, MF417586) showed that they differed from the SSA1-SG3 sequence JQ286482 by only three C/T substitutions that resulted in 0.56% nucleotide difference (Suppl. Fig. 2A). The basis for suggesting SSA1-SG5 and SSA1-SG3 to be different ‘subgroups’ within the SSA1 species was therefore unclear. For example, sequences with similar (Suppl. Fig. 2B) or with greater (Suppl. Fig. 2C) numbers of nucleotide substitutions were placed within same subgroups, or potentially as the same species^31^ (Suppl. Fig. 2D).

### Species delimitation and introgression

**Figure 1:**
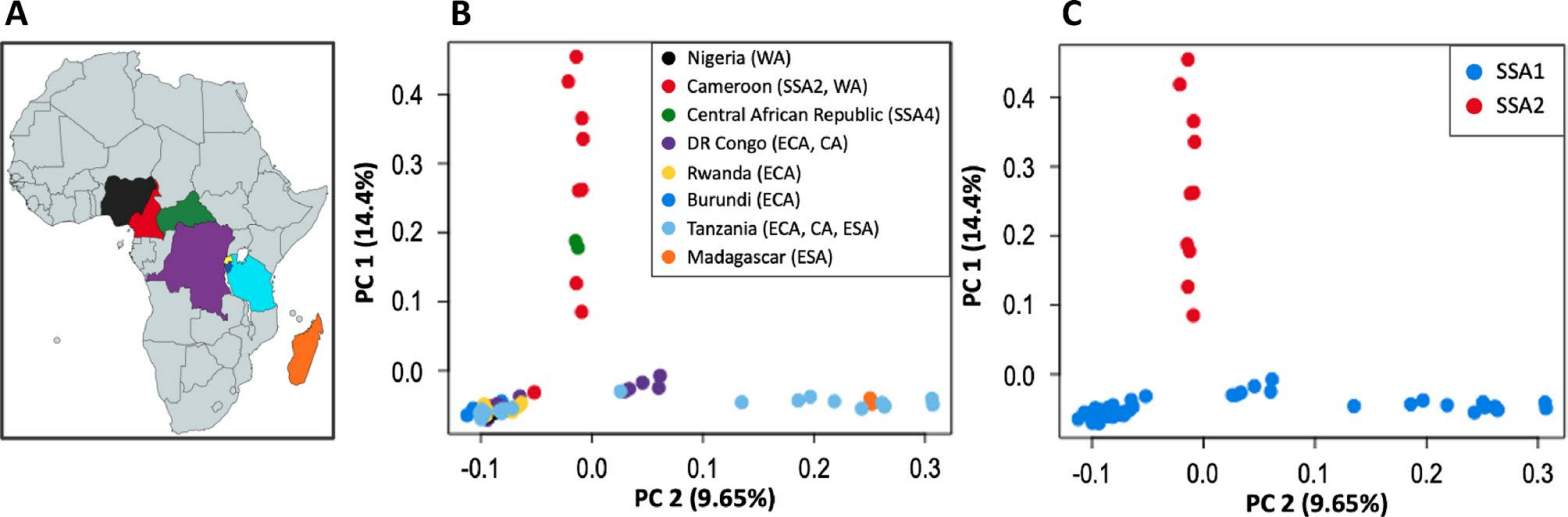
Sampling sites and principal component analysis of SSA *Bemisia* samples. Principal component analysis (PCA) was based on 14,358 genome-wide SNPs across the 62 sub-Saharan African cassava *Bemisia* samples from eight sub-Saharan countries. (**A**) African countries where whitefly individuals were sampled from. Country names and colours as (**B**). African map created using mapchart <mapchart.net>. (**B**) Country of origins for these SSA1 and SSA2 *Bemisia* species are shown by unique colour codes. Parentheses after country names are the ‘genetic groups’ as proposed by Wosula et al.^38^. (**C**) PCA showing clustering of individuals by their SSA1 or SSA2 species status from this study’s re-analyses of genome-wide SNPs.

The genome-wide SNP-based Principal Component Analysis (PCA) discriminated between SSA1 and SSA2 sampled from eight sub-Saharan African nations (Fig. 1A), suggesting that SSA1 and SSA2 are genetically distinct and likely to be different species as supported by mating studies^31,34^. Samples from SSA4 clustered with SSA2 (Fig. 1B,C), suggesting that mtCOI-based SSA4 species identification was likely misidentification of SSA2 due to NUMTs^29^. Removal of NUMTs resolved the mtCOI discrepancies, and reanalyses of genome-wide SNP data provided insights into SSA1-species-wide population substructure, identified likely geographic contact zones, and signatures of infra- and inter-species hybridization.

**Figure 2:**
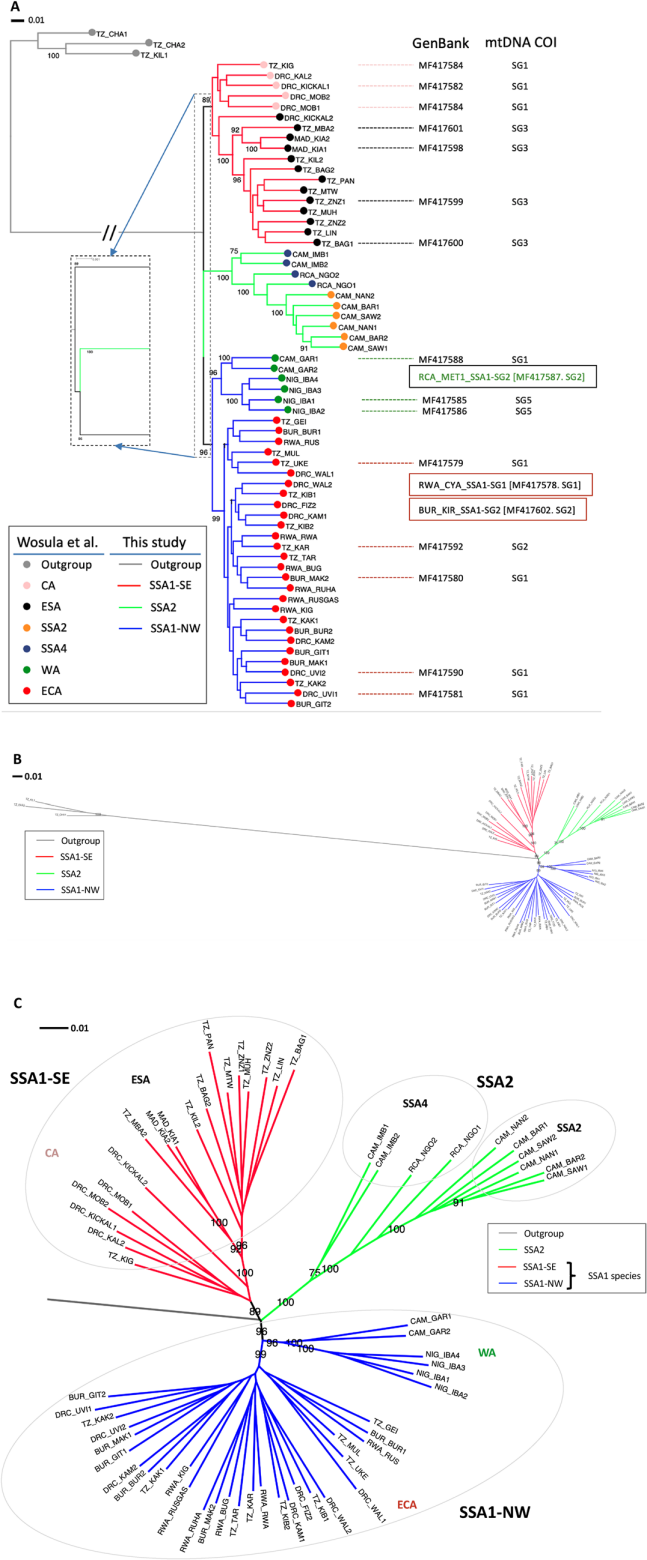
Phylogeny of SSA populations. (**A**) RAxML phylogeny with 1000 bootstrap replications based on 14,358 nextRAD genome-wide SNPs from 62 SSA *Bemisia* individuals and three ‘non-cassava individuals’^38^ as outgroup. Branch colour schemes (red, green, blue) are as used in Admixture analysis (see Fig. 3). Sample names and branch tip colour schemes (filled circles) followed Wosula et al.^38^, and where available, mtCOI GenBank accession numbers corresponding to selected samples, and their proposed mtCOI SSA1-subgroups (SG)^13^ status (i.e., SG1, SG2, SG3, SG5^44^) are also listed. Individuals that have partial mtCOI gene sequence but have been excluded from the nextRAD genome-wide SNPs phylogeny analysis due to poor SNP data coverage are boxed. The *Bemisia* SSA2-SSA4 clade is indicated by green branches, and the SSA lineages as proposed by Wosula et al.^38^ [i.e., CA (pink), ESA (black), WA (green), ECA (red)] are here identified as belonging to the SSA1 species and together, made up the ‘SSA1-SE’ and ‘SSA1-NW’ putative sub-species. Note that only nodes with ≥ 75% bootstrap support are shown. Enlarged section of branch breaks separating SSA1-NW, SSA1-SE, and SSA2 are shown by the insert box. The long branch length (0.43) separating the outgroups and the SSA1/SSA2 clades has been shorten as indicated by ‘//’. (**B**) Radial phylogeny with sample names and lineage colours as for (**A**). (**C**) Detail of the RAxML phylogeny in (**B**) showing only the SSA1 (i.e., represented by all ‘SE’ and ‘NW’ individuals) and SSA2 (represented by SSA2 and ‘SSA4’ individuals) clades. Proposed genetic groups for the SSA1 species by Wosula et al.^38^ are also indicated (i.e., ‘CA’ and ‘ESA’, and ‘WA’ and ‘ECA’ within the putative SSA1-SE and SSA1-NW sub-species, respectively). Country codes are: Tanzania (TZ), Democratic Republic of Congo (DRC), Madagascar (MAD), Cameroon (CAM), Central African Republic (RCA), Nigeria (NIG), Rwanda (RWA), Burundi (BUR).

Maximum-Likelihood (ML) phylogeny inferred using RAxML based on 14,358 genome-wide SNPs (Fig. 2A) also supported the PCA result and broadly divided these 62 SSA individuals into three clusters that consisted of: (i) SSA2 (i.e., individuals previously identified as SSA4 or SSA2 by mtCOI; 100% node confidence), (ii) SSA1-SE (i.e., ‘SSA-CA/SSA-ESA’ populations; node confidence 89%), and (iii) SSA1-NW (i.e., ‘SSA-ECA/SSA-WA’ populations; node confidence 96%). The phylogenomic analysis supported the SSA1 and SSA2 species status as consistent with results from partial mtCOI gene sequences (Fig. 2B,C). Two clades within SSA1, denoted as SSA1-SE (South-Eastern) and SSA1-NW (North-Western) (Fig. 2C), respectively, was evident, reflecting the geographic distribution of the populations identified within them (SSA1-SE individuals from DR Congo, Tanzania, Madagascar; SSA1-NW samples from Cameroon, Nigeria, DR Congo, Rwanda, Burundi, Tanzania). Within SSA1-NW, the ‘SSA-ECA’ and ‘SSA-WA’ populations^38^ are clustered separately with 99% and 96% confidence values. Within SSA1-SE, the ‘SSA-ESA’ and ‘SSA-CA’ populations^38^ are partially resolved, sharing only 34% confidence and suggesting insufficient genomic evidence to support their separate genetic group classification^38^.

**Figure 3:**
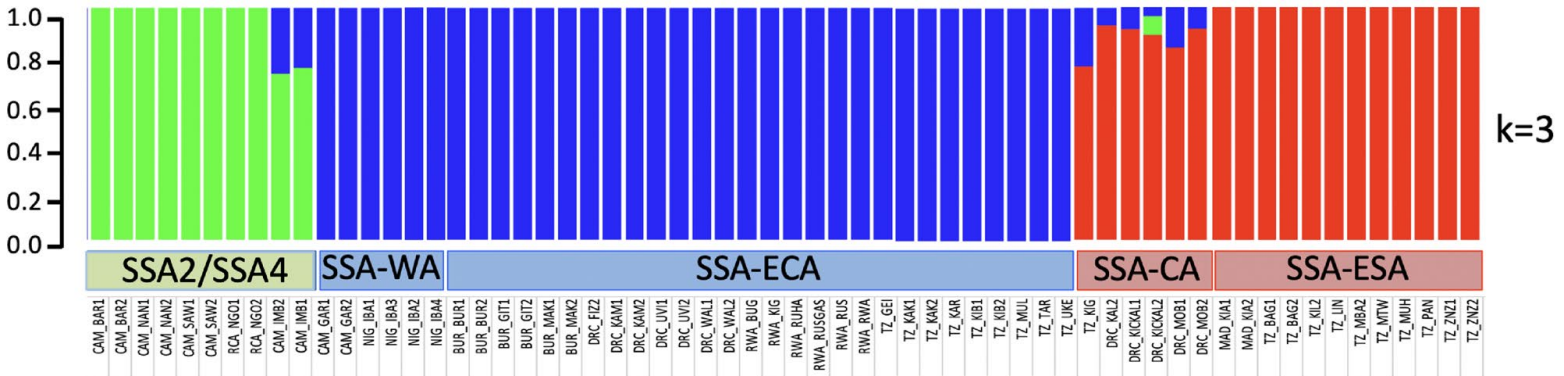
Admixture analysis at K = 3 of 62 individuals of sub-Saharan African *Bemisia* cryptic species based on 14,358 SNPs. Individuals were clustered into the SSA2 species group, as well as two distinct clusters (red, blue) that belonged to the SSA1 species. The two SSA1 clusters in the Admixture panel included the SSA-WA and SSA-ECA groups (34 blue individuals; blue = SSA1-NW), and six SSA-CA (blue-red/blue-red-green) and 12 SSA-ESA (red; SSA1-SE) individuals. Evidence of introgression between the red and blue SSA1 clusters were predominantly detected in SSA-CA individuals. Three SSA2/SSA1 inter-specific hybrids (CAM_IMB2_SSA4, CAM_IMB1_SSA4, DRC_KICKAL2_CA) were also detected. Note: Figure colour scheme matches the phylogeny branch colours of Fig. 2A–C.

Admixture analysis identified three genetic clusters (Fig. 3) that followed the partial mtCOI gene-based species diagnosis (see also Suppl. Fig. 3), whereby the SSA2 species was clearly shown to be different from the SSA1 species. The SSA1 species was further shown to consist of two distinct genetic backgrounds corresponding to SSA1-NW and SSA1-SE (containing ‘blue’ SSA-WA and SSA-ECA, and ‘red’ SSA-CA and SSA-ESA, respectively, as defined by Wosula et al.^38^). Some SSA2 and all the SSA1-CA samples showed evidence for low levels of admixture.

Interestingly, *F*_st_ analysis (Table 1) supported SSA1-NW and SSA1-SE as potential putative sub-species of SSA1, with intermediate *F*_st_ estimates observed for all pair-wise comparisons at the inter-specific (i.e., SSA2 vs. SSA1; *F*_st_ = 0.286–0.334) level. This was particularly evident for SSA1-NW versus the SSA1-SE ‘ESA’ population, with *F*_st_ of 0.226–0.243. *F*_st_ differentiation was less clearly defined for the SSA-CA population, whose *F*_st_ scores versus SSA1-NW were closer to those between populations within the two major SSA1 groups. The overall differentiation between SSA1-NW and SSA1-SE suggested likely existence of putative sub-species within SSA1, the observations concerning the SSA-CA population suggest gene flow between neighbouring populations. Treatment of SSA1 as single species against the SSA2/SSA4 species showed significant population substructure (*F*_st_ range 0.286–0.332). This limited gene flow between SSA2/SSA4 and the SSA1 (i.e., SSA-WA, SSA-ECA, SSA-CA. SSA-ESA) supported their separate species status. Treatment of the four SSA1 populations as belonging to two well-defined groups (i.e., SSA-NW, SSA-SE) akin to ‘putative sub-species’, showed that SSA-WA and SSA-ECA overall shared intermediate levels of gene flow with the SSA-ESA population. *F*_st_ estimates are lowest between the SSA-CA population and the SSA-WA, SSA-ECA, and SSA-ESA populations, suggesting greater gene flow at the contact zone.

**Table 1:** Population substructure based on the *F*-statistics (*F*_st_) of Weir and Cockerham^45^.

Species	SSA2	SSA1			
Sub-species		SSA-NW		SSA-SE	
Populations	SSA2/SSA4	SSA-WA	SSA-ECA	SSA-CA	SSA-ESA
SSA2/SSA4					
SSA-WA	0.291				
SSA-ECA	0.334	0.171			
SSA-CA	0.286	0.166	0.158		
SSA-ESA	0.332	0.243	0.226	0.160	

**Figure 4:**
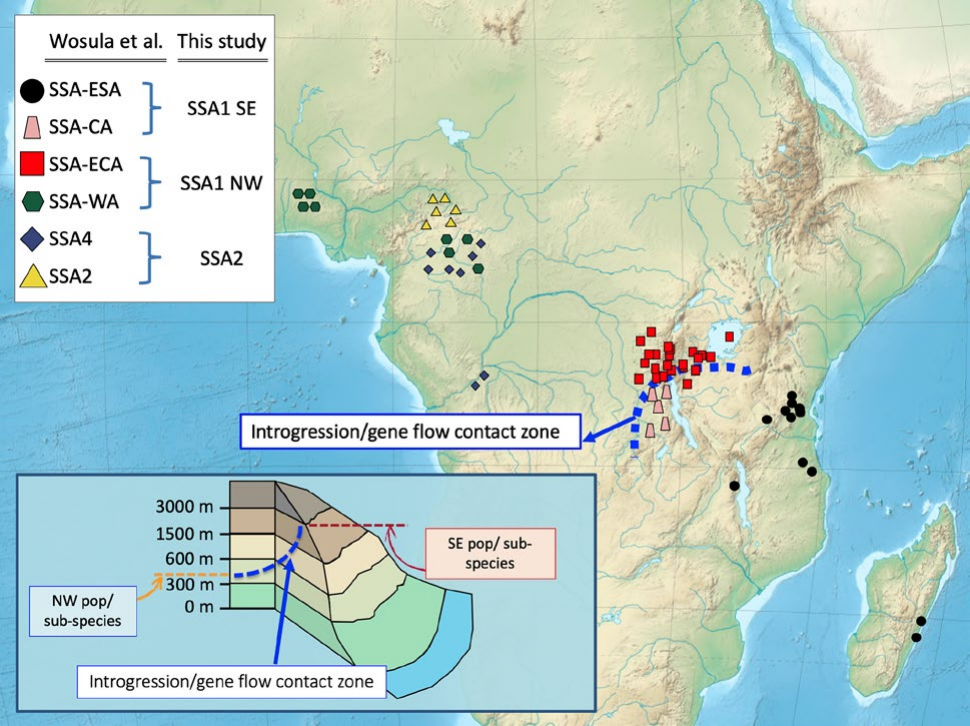
A map showing sampling sites of the SSA *Bemisia* cryptic species. The sample colour schemes and sampling sites followed Fig. 6B of Wosula et al.^38^ (green: SSA-WA; red: SSA-ECA; pink: SSA-CA; black: SSA-ECA; Yellow: SSA2; blue: SSA4). Note: The SSA4 species is likely NUMT artefacts of the SSA2 species (refer to main text). The African topological map colour represents elevation from sea-level (see insert map of non-incremental approximation of elevation). The gene flow contact zone between populations on the NW–SE transect is indicated by the thick blue dashed line. Map modified and prepared using Microsoft PowerPoint for Mac, Version 16.39 (20071300). Sourced: https://commons.wikimedia.org/wiki/File:Africa_topography_map.png.

Within the putative SSA1-NW sub-species, the SSA-WA population is predominantly found in the lowlands (*ca*. 300–1500 m), whereas the SSA-ECA/SSA-CA populations were found at higher altitudes (from *ca*. 600–≥ 1500 m). Individuals belonging to the SSA-ESA group were predominantly from coastal landscape of Tanzania and Madagascar (Fig. 4). A gene flow contact zone was detected at regions represented by five DR Congo individuals and one Tanzanian individual (i.e., the SSA-CA population). Inter-specific hybridization was detected in two SSA2 individuals (Fig. 3, green/blue genetic backgrounds) and in one DR Congo individual from SSA-CA (red/green/blue in Fig. 3).

**Figure 5:**
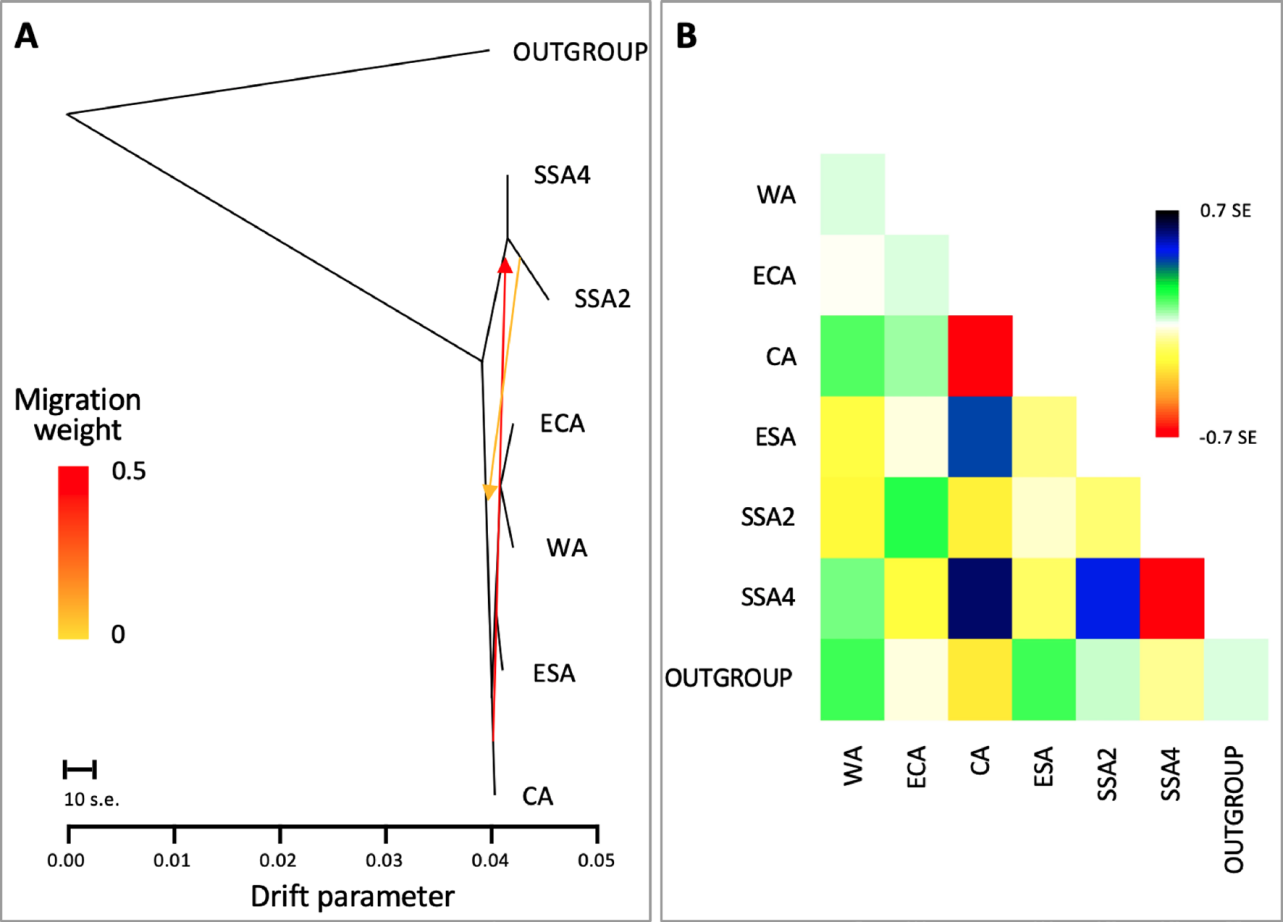
Gene flow between SSA species. (**A**) TreeMix ML tree with two migration edges (m = 2), showing strong gene flow from SSA1 to SSA2/SSA4 (red arrow), and weaker gene flow from SSA2 to SSA1. Clustering between ECA and WA, and between ESA and CA, provided support for their close relationships that could potentially indicate their respective putative sub-species status (i.e., SSA1-NW, SSA1-SE, respectively). Two basal branches leading to SSA1 populations (i.e., ECA, WA, ESA, CA) and SSA2/SSA4 further supported the populations analysed as consisted of two species (i.e., SSA1, SSA2). The scale bar indicates ten times the average standard error (s.e.). (**B**) The residual fit was plotted from the ML tree, which is the covariance matrix between each pair of populations. Residuals above zero are an indication of the populations most likely to be admixed.

The Treemix ML tree supported the SSA4 species as part of SSA2, and the migration edges (m = 2) revealed gene flow in both directions, between SSA1 and SSA2 species from central Africa (i.e., SSA-CA), and supports detection of hybrid individuals identified in the Admixture analysis (Fig. 3). The weight of the migration edge going from SSA-CA to SSA2 is also higher than the one going the opposite direction (Fig. 5). TreeMix ML tree also showed a lack of branch depth between each cluster of SSA2/SSA4, ECA/WA, and CA/ESA, thereby provided further support that entities within the three clusters were identical.

## Discussion

We combined genome-wide SNPs and mtCOI markers to gain evolutionary, ecological and population genomic insights into the sub-Saharan African cassava *Bemisia* whitefly species complex. By taking into consideration the impact of NUMTs, we showed no conflict in the same nuclear and mtCOI dataset in defining *Bemisia* species status. A thorough filtering and reanalysis of genome-wide SNP markers also detected signatures of incipient speciation and admixture in the *Bemisia* SSA1 species. While various evolutionary genetic studies based on the partial mtCOI gene have proposed the presence of SSA1 species subgroups^13,46^, we caution against the use of the mitochondrial ‘subgroup’ classification based on genome-wide SNP markers. Mating experiments were inconclusive in separating subgroups of SSA1 especially between SG1 and SG2^31^, while for the first time, genome-wide SNP loci showed that the SSA1 species could potentially be defined as to consist of a north-western (NW) and a south-eastern (SE) putative sub-species, with a contact zone separated by the eastern highland and central/western lowland regions.

Wosula et al.^38^ adopted the nextRAD genome-wide SNP approach first demonstrated by Elfekih et al.^10,47–49^ for *Bemisia* species to investigate gene flow patterns and diversity in the SSA1, SSA2, and SSA4 African cassava *Bemisia* whitefly species complex, but overlooked the impact of NUMTs^10,32,35^ in published *B. tabaci* phylogenetic studies based on partial mtCOI sequences. This led to the conclusion that the mtCOI gene lacked efficacies at differentiating African *Bemisia* cryptic species. Increasingly, reanalyses of the *B. tabaci* cryptic species complex partial mtCOI gene dataset have detected the presence of significant NUMTs and pseudogenes^3,29,35^. These studies highlighted on-going challenges associated with species delimitation in the cryptic *B. tabaci* species complex. Similar to MEAM2^28,50^, our analysis supported that the SSA4^42^ was likely NUMT artefacts as previously proposed^29^. The misidentification of SSA4 underpinned all subsequent genomic interpretations of gene flow and evolutionary ecology in the cassava SSA *Bemisia* whitefly species^38^.

Significant genetic diversity in the *B. tabaci* species complex can obscure species/putative sub-species identification. For instance, high genetic diversity in the MED species based on nextRAD SNP data has been reported^10^; however, the MED-ASL as a separate species was only further supported by including also complete mitogenome, mating experiments^3^, and host preference studies^5^. Genomics and mating experiments similar to the MED-ASL studies^3,5^ showed that the SSA1-SG1 and SG2 were likely the same species, with SG3 potentially being different^31^ (i.e., the ‘WA’ clade; Fig. 2B,C). Similar studies are needed to provide additional biological support for various SSA *Bemisia* species subgroups^51^ including the putative SSA1-NW and SSA1-SE sub-species.

In developing the DNA barcoding approach, Hebert et al.^52^ showed that the COI gene is overall effective for species level delimitation due to the associated nucleotide substitutional rates for this gene (but see Hanemaaijer et al.^53^), while for fine-scale identification such as within species level, the use of genes with greater mutational rates were recommended. Partial mtCOI gene therefore lacks the power to differentiate between evolutionary very closely related cryptic species status, for which a genomic approach should be considered^3,31^. Subgroup classification within the SSA1 African cassava *Bemisia* whitefly species based on the mtDNA gene might be possible, provided that a gene region with an appropriate substitutional rate is identified. Nevertheless, there is currently no evidence for maternal lineage-based patterns to population dispersal behaviour, host adaptation, and fitness cost differentiation in the *B. tabaci* complex and the African cassava *Bemisia* species complex, and therefore the subgroup delimitation based on the partial mtCOI gene is not justified.

The SSA1-SG1, representing the major SSA1 mtCOI subgroup, was previously implicated in the CMD pandemic in East Africa^13^. Wosula et al.^38^ reclassified SSA1-SG1 individuals (e.g., SSA1-SG3, SSA1-SG5^44^) into three SNP-based groups of SSA-ECA, SSA-CA, and SSA-WA, due to discrepancies between the 657 bp mtCOI partial gene and nuclear SNP markers. Based on the same nuclear and mitochondrial dataset, we showed that while separate phylogenetic signals were detected for the ‘WA’ and ‘ECA’ populations, a strong genetic difference between the ‘CA’ and ‘ESA’ populations was lacking, and we therefore advocate following established good practice^51^ to refrain prematurely adopting other nomenclature (i.e., those proposed by Wosula et al.^38^).

Although refined higher-level species status could help resolve virus transmission capacity and provide insights into understanding pest introduction pathways^11,54^, there is currently insufficient evidence in African cassava *Bemisia* whiteflies to link either the WA/ECA or CA/ESA populations to historical CMD outbreaks. The *B. tabaci* SSA2 (i.e., ‘Ug2’^33^) was assumed to be associated with the 1990s CMD epidemic, mainly due to its geographic distribution in relation to the front of the epidemic^55^. However, transmission underpinning outbreaks of CMD in the sub-Saharan Africa region is complex, with one factor being the sharing and propagation of diseased cassava cuttings between farmers. There was a recombination event between two geminiviruses that produced a more virulent hybrid^56^, however we have little historical information about how the viruses interacted with the different *Bemisia* cryptic species in terms of disease dynamics^57^. Evidence for bidirectional interspecific hybridization detected between SSA1 and SSA2 in this study, therefore may have implications to understanding the evolutionary genetics associated with plant virus transmission capacity by the cassava SSA *Bemisia* whitefly species complex. The role of the SSA2 species in the CMD epidemic nevertheless remained unclear, with the SSA2’s habitat range (east and west Africa) overlapping the SSA1 species^22,38,55,58–62^. The exact relationship between SSA1/SSA2 and outbreaks of CMD^63^ should be re-visited, taking into consideration current genomic evidence for the presence of SSA1/SSA2 hybrids. Furthermore, vector competencies of other common cassava whitefly species (e.g., *B. afer*) to different cassava viruses also need further investigation, an ideally include a genomic component.

*B. tabaci *sensu lato on cassava in sub-Saharan Africa is a species complex going through a continuous diversification process. The putative SSA1-SE sub-species that the Madagascan (SSA-ESA) and Tanzanian (SSA-CA) populations belong to are geographically isolated from the Nigerian (SSA-WA), DR Congo, and Burundi (SSA-ECA) populations with a hybrid zone between Tanzania, Uganda and DR Congo. The East African Rift Valley is a major geographical barrier to gene flow and provides the environment to promote incipient speciation and to enable recent radiations to thrive and evolve^64^, and could underpin incipient allopatric speciation in the SSA1. The “rift effect” acting as a gene flow barrier has been described in other insects (e.g., the maize stalk borer *Busseola fusca*^65^, the malaria vector mosquitoes *Anopheles gambiae*^66,67^) and vertebrates (e.g., birds^68^, frogs^69^). Specifically, the western branch of the Rift Valley, i.e., the Albertine Rift covering part of Tanzania, Burundi, Rwanda, Uganda and DR Congo, is a mountain chain situated at 1500–3500 m above sea level (Fig. 4) formed by volcanic activity since the plio-pleistocene. In addition to volcanic activities, climate change during this glacial period led to habitat fragmentation that promoted species diversification in the newly formed biogeographical zones^70^. Species diversification in the African *Bemisia* whitefly cryptic species system has also been explained by host plant associations and their ability to feed on diverse host plants and acquiring new ones^71^. Using RNAseq, Malka et al.^72^ provided insights into how the variation in detoxification gene expression levels might have conferred plasticity that likely also contributed to species diversification.

Finally, we note that our genome-wide SNP phylogenetic analysis showed weak support (bootstrap value 34%, data not shown) for closer evolutionary relationship between SSA2 and SSA1-NW, than between the two putative SSA1 sub-species. This closer phylogenetic relationship between SSA2 and SSA1-NW is likely to be unreliable given the weak bootstrap support, greater gene flow patterns between the SSA1 sub-species (Table 1), and the congruence of species status based on mtCOI molecular diagnostics. our study therefore further highlights the need for careful selection of outgroups that takes into consideration the evolutionary biological questions to be addressed (e.g., whether the target species were more closely related (i.e., between SSA1, SSA2, SSA3, SSA6, SSA9)^29^ or evolutionary diverged (i.e., between SSA1 and AsiaII_1)^29^). Data obtained for this population genomic reanalysis involved only limited number of target and outgroups individuals and species that represented significant evolutionary divergent lineages from the SSA1/SSA2 species^38^. The challenge of obtaining sufficient sequencing coverage that is necessary for filtering to obtain a robust set of genome-wide SNP markers for the *Bemisia* cryptic species complex^10,47–49^ known to have a large and complex genome organization^73,74^ will likely further enhance and perpetuate their on-going status as one of the world’s most damaging and challenging group of agricultural insect pest complex.

## Material and methods

### mtCOI data handling

The mtCOI sequences of Wosula et al.^38^ were downloaded from GenBank (MF417578-MF417602), including all published reference sequences used to identify the SSA1 ‘genetic groups’. These partial mtCOI sequences were imported into Geneious R11.1 and aligned with reference sequences (Suppl. Fig. 1) using the MAFFT alignment program^75^ with the following settings (Algorithm option set at ‘Auto’; Scoring matrix at 200PAM/K = 2; Gap open penalty and Offset values at 1.53 and 0.123, respectively). We examined all sequences for: (i) insertion/deletions (indels), (ii) presence of premature stop codons, and (iii) presence of unexpected amino acid substitutions at evolutionary conserved regions in the partial COI gene region^29^ as signatures of pseudogenes (NUMTs).

### nextRAD data processing

The raw data from nextRAD sequencing^38^ was accessed from GenBank (SRP103541). The fastq sequences were screened using FastQC v.0.11.4 for quality control. The reads were trimmed by quality and reads longer than 151 bp was trimmed to this length using Trimmomatic^76^ version 0.36.

### Mapping quality

We mapped the reads from each individual sample to the *B. tabaci* MEAM1 genome^73^ using Burrows-Wheeler Aligner (BWA) v.0.7.12^77^. The alignments used the BWA-MEM algorithm with default settings. The SAM files were converted to BAM output using Samtools^78^ v. 1.3.1. The BAM files were subsequently sorted, indexed and checked for quality and mapping percentages per scaffold.

### SNP genotyping

SNPs were first called using a de novo approach in PyRAD v. 0.9^79^. PyRAD is a pipeline designed for RADseq datasets that aims to capture variation at the species/clade level. It allows clustering of highly divergent sequences and takes into consideration indel variation. We also performed SNP calling in PyRAD, using reads mapped to the MEAM1 reference genome^73^.

### Genetic clusters and tests for introgression

Genetic clusters within the dataset that consists of 62 SSA samples were investigated by Principal Component Analysis (PCA) using the SNPRelate R package^80^. We used ADMIXTURE v.1.3.0^81^ on the 62-sample dataset to identify genetic clusters and estimate the genetic ancestry of the SSA samples. We ran the program with K ancestral clusters varying from 1 to 10 with each ‘K’ value repeated 100 times. A cross-validation test was performed to determine the optimal K value.

### Phylogenetic analyses

The topology of the phylogenetic relationships between individuals/populations/sub-species and species within the dataset (62 SSA samples and three outgroup individuals (TZ_CHA1, TZ_CHA2, TZ_KIL1)) was examined using a maximum likelihood (ML) approach. The SSA species status were first identified using the mtCOI marker, then, a phylogeny was reconstructed using the nextRAD genome-wide SNPs excluding samples with low genotype quality (mapping quality < 10) to minimize biases that could potentially be introduced by missing data. We performed five replicates of phylogenetic reconstruction in RAxML v.7.2.8^82^ using the GTR substitution model and GTRGAMMA as the GAMMA model of rate heterogeneity, with 1000 bootstrap replications. TreeMix^83^ v. 1.12 was used to identify genetic mixing, the history of population splits and admixture patterns. We ran simulations in order to infer the best-fit scenario for the genetic relationships between these cassava *Bemisia* whitefly populations involving up to 5 migrations events with 100 bootstrap replications (e.g., based on previous studies using this type of analysis^49,84,85^. The likelihoods were compared using the following command in R: tail -n 1 Data/data.frq.strat.tree.*.*.llik | cut -d \: -f 2 > Data/data. The decision to select the number of migration m = 2 is based on the residual fit matrix generated by the TreeMix program. For each migration (m = 0, m = 1…, m = 5), we generate a residual plot. When this plot is saturated at a given m, the best fit scenario and the best-fit ML tree that corresponds to the specific m number are obtained. If it is m = 2, then any number bigger than 2 would not add any more information. Programming scripts used in this study are available as Supplemental Data 1–7.

## Supplementary Information

Supplementary Information 1.

Supplementary Information 2.

Supplementary Information 3.

Supplementary Information 4.

Supplementary Information 5.

Supplementary Information 6.

Supplementary Information 7.

Supplementary Information 8.

## References

[1] Hebert PD, Penton EH, Burns JM, Janzen DH, Hallwachs WT (2004). species in one: DNA barcoding reveals cryptic species in the neotropical skipper butterfly *Astraptes fulgerator*. Proc. Natl. Acad. Sci. U.S.A..

[2] Saez AG, Lozano E (2005). Body doubles. Nature.

[3] Vyskočilová S, Tay WT, van Brunschot S, Seal S, Colvin J (2018). An integrative approach to discovering cryptic species within the *Bemisia tabaci* whitefly species complex. Sci. Rep..

[4] Liu SS (2007). Asymmetric mating interactions drive widespread invasion and displacement in a whitefly. Science.

[5] Vyskocilova S, Seal S, Colvin J (2019). Relative polyphagy of "Mediterranean" cryptic *Bemisia tabaci* whitefly species and global pest status implications. J. Pest Sci..

[6] Behere GT, Tay WT, Russell DA, Batterham P (2008). Molecular markers to discriminate among four pest species of *Helicoverpa* (Lepidoptera: Noctuidae). Bull. Entomol. Res..

[7] Elfekih S, Tay WT, Gordon K, Court LN, De Barro PJ (2018). Standardized molecular diagnostic tool for the identification of cryptic species within the *Bemisia tabaci* complex. Pest Manag. Sci..

[8] Walsh TK (2019). Mitochondrial DNA genomes of five major *Helicoverpa* pest species from the Old and New Worlds (Lepidoptera: Noctuidae). Ecol. Evol..

[9] Anderson CJ, Tay WT, McGaughran A, Gordon K, Walsh TK (2016). Population structure and gene flow in the global pest, *Helicoverpa armigera*. Mol. Ecol..

[10] Elfekih S (2018). Genome-wide analyses of the *Bemisia tabaci* species complex reveal contrasting patterns of admixture and complex demographic histories. PLoS ONE.

[11] Anderson CJ (2018). Hybridization and gene flow in the mega-pest lineage of moth, *Helicoverpa*. Proc. Natl. Acad. Sci. U.S.A..

[12] FAOSTAT. http://www.fao.org/faostat/en/#data/QC/visualize (2017).

[13] Legg JP (2014). Spatio-temporal patterns of genetic change amongst populations of cassava *Bemisia tabaci* whiteflies driving virus pandemics in East and Central Africa. Virus Res..

[14] Patil BL, Fauquet CM (2009). Cassava mosaic geminiviruses: Actual knowledge and perspectives. Mol. Plant Pathol..

[15] Macfadyen S (2018). Cassava whitefly, *Bemisia tabaci* (Gennadius) (Hemiptera: Aleyrodidae) in East African farming landscapes: A review of the factors determining abundance. Bull. Entomol. Res..

[16] Minato N (2019). Surveillance for Sri Lankan cassava mosaic virus (SLCMV) in Cambodia and Vietnam one year after its initial detection in a single plantation in 2015. PLoS ONE.

[17] Wang HL (2016). First Report of Sri Lankan cassava mosaic virus Infecting Cassava in Cambodia. Plant Dis..

[18] De Barro PJ, Liu SS, Boykin LM, Dinsdale AB (2011). *Bemisia tabaci*: A statement of species status. Annu. Rev. Entomol..

[19] Hopkinson J (2020). Insecticide resistance status of *Bemisia tabaci* MEAM1 (Hemiptera: Aleyrodidae) in Australian cotton production valleys. Austral Entomol..

[20] Hadjistylli M, Roderick GK, Gauthier N (2015). First report of the Sub-Saharan Africa 2 species of the *Bemisia tabaci* complex in the Southern France. Phytoparasitica.

[21] Lee W, Park J, Lee GS, Lee S, Akimoto S (2013). Taxonomic status of the *Bemisia tabaci* complex (Hemiptera: Aleyrodidae) and reassessment of the number of its constituent species. PLoS ONE.

[22] Mugerwa H (2018). African ancestry of New World, *Bemisia tabaci*-whitefly species. Sci. Rep..

[23] Martin JH (1987). An identification guide to common whitefly pest species of the world (Homopt Aleyrodidae). Int. J. Pest Manag..

[24] Martin JH, Mound LA (2007). An annotated check list of the world's whiteflies (Insecta: Hemiptera: Aleyrodidae). Zootaxa.

[25] Mound LA (1963). Host-correlated variation in *Bemisia tabaci* (Gennadius). Proc. R. Entomol. Soc. Lond..

[26] Tay WT (2017). Novel molecular approach to define pest species status and tritrophic interactions from historical *Bemisia* specimens. Sci. Rep..

[27] Tay WT, Evans GA, Boykin LM, De Barro PJ (2012). Will the real *Bemisia tabaci*please stand up?. PLoS ONE.

[28] Dinsdale A, Cook L, Riginos C, Buckley YM, De Barro P (2010). Refined global analysis of *Bemisia tabaci* (Hemiptera: Sternorrhyncha: Aleyrodoidea: Aleyrodidae) mitochondrial cytochrome oxidase 1 to identify species level genetic boundaries. Ann. Entomol. Soc. Am..

[29] Kunz D, Tay WT, Elfekih S, Gordon KHJ, De Barro PJ (2019). Take out the rubbish - Removing NUMTs and pseudogenes from the *Bemisia tabaci*cryptic species mtCOI database. bioRxiv..

[30] Wongnikong W, van Brunschot SL, Hereward JP, De Barro PJ, Walter GH (2020). Testing mate recognition through reciprocal crosses of two native populations of the whitefly *Bemisia tabaci* (Gennadius) in Australia. Bull. Entomol. Res..

[31] Mugerwa H, Wang H-L, Sseruwagi P, Seal S, Colvin J (2020). Whole-genome single nucleotide polymorphism and mating compatibility studies reveal the presence of distinct species in sub-Saharan Africa *Bemisia tabaci* whiteflies. Insect Sci..

[32] Delatte H (2005). A new silverleaf-inducing biotype Ms of *Bemisia tabaci* (Hemiptera: Aleyrodidae) indigenous to the islands of the south-west Indian Ocean. Bull. Entomol. Res..

[33] Boykin LM, Savill A, De Barro P (2017). Updated mtCOI reference dataset for the *Bemisia tabaci* species complex. F1000Research.

[34] Liu SS, Colvin J, De Barro PJ (2012). Species concepts as applied to the whitefly *Bemisia tabaci* systematics: How many species are there?. J Integr Agr.

[35] Tay WT (2017). The trouble with MEAM2: Implications of pseudogenes on species delimitation in the globally invasive *Bemisia tabaci* (Hemiptera: Aleyrodidae) cryptic species complex. Genome Biol. Evol..

[36] Kunz D (2019). Draft mitochondrial DNA genome of a 1920 Barbados cryptic *Bemisia tabaci* 'New World' species (Hemiptera: Aleyrodidae). Mitochondrial DNA B.

[37] Paini DR (2016). Global threat to agriculture from invasive species. Proc. Natl. Acad. Sci. U.S.A..

[38] Wosula EN, Chen WB, Fei ZJ, Legg JP (2017). Unravelling the genetic diversity among cassava *Bemisia tabaci* whiteflies using NextRAD sequencing. Genome Biol. Evol..

[39] Thresh JM, Fargette D, Otim-Nape GW (1994). Effects of African cassava mosaic geminivirus on the yield of cassava. Trop. Sci..

[40] Legg J (2014). A global alliance declaring war on cassava viruses in Africa. Food Secur..

[41] Legg JP (2014). Biology and management of *Bemisia* whitefly vectors of cassava virus pandemics in Africa. Pest Manag. Sci..

[42] Berry SD (2004). Molecular evidence for five distinct *Bemisia tabaci* (Homoptera: Aleyrodidae) geographic haplotypes associated with cassava plants in sub-Saharan Africa. Ann. Entomol. Soc. Am..

[43] Mugerwa H, Rey MEC, Tairo F, Ndunguru J, Sseruwagi P (2019). Two sub-Saharan Africa 1 populations of *Bemisia tabaci* exhibit distinct biological differences in fecundity and survivorship on cassava. Crop Prot..

[44] Ghosh S, Bouvaine S, Maruthi MN (2015). Prevalence and genetic diversity of endosymbiotic bacteria infecting cassava whiteflies in Africa. BMC Microbiol..

[45] Weir BS, Cockerham CC (1984). Estimating F-statistics for the analysis of population-structure. Evolution.

[46] Ghosh S, Bouvaine S, Richardson SCW, Ghanim M, Maruthi MN (2018). Fitness costs associated with infections of secondary endosymbionts in the cassava whitefly species *Bemisia tabaci*. J. Pest Sci..

[47] Elfekih, S. *et al.* Evolutionary genomics of *Bemisia tabaci* and characterization of its endosymbiont metacommunities using nextRAD sequencing. *International Plant and Animal Genome Asia, Singapore* 23–25 July 2015 (2015).

[48] Elfekih, S. *et al.* Genome-wide SNPs Decipher Global Incursion pathways in the *Bemisia tabaci* species complex. *International Plant and Animal Genome Conferences* San Diego, 9–13 January 2016 (2016)*.*

[49] Elfekih, S. *et al.* Genome-wide scans unravel fine-scale invasion routes in the *Bemisia tabaci* species complex. *2nd International Whitefly Symposium, Arusha, Tanzania. p38,* 14–19 February 2016 (2016).

[50] Boykin LM, Bell CD, Evans G, Small I, De Barro PJ (2013). Is agriculture driving the diversification of the *Bemisia tabaci* species complex (Hemiptera: Sternorrhyncha: Aleyrodidae)? Dating, diversification and biogeographic evidence revealed. BMC Evol. Biol..

[51] Boykin LM (2018). Review and guide to a future naming system of African *Bemisia tabaci* species. Syst. Entomol..

[52] Hebert PDN, Cywinska A, Ball SL, DeWaard JR (2003). Biological identifications through DNA barcodes. Proc. R. Soc. B Biol. Sci..

[53] Hanemaaijer MJ (2019). Mitochondrial genomes of *Anopheles**arabiensis*, *An. gambiae* and *An. coluzzii* show no clear species division [version 2; peer review: 2 approved]. F1000Research.

[54] Tabachnick WJ (1996). Culicoides variipennis and bluetongue-virus epidemiology in the United States. Annu. Rev. Entomol..

[55] Legg JP, French R, Rogan D, Okao-Okuja G, Brown JK (2002). A distinct *Bemisia tabaci* (Gennadius) (Hemiptera: Sternorrhyncha: Aleyrodidae) genotype cluster is associated with the epidemic of severe cassava mosaic virus disease in Uganda. Mol. Ecol..

[56] Colvin J, Omongo CA, Maruthi MN, Otim-Nape GW, Thresh JM (2004). Dual begomovirus infections and high *Bemisia tabaci* populations: Two factors driving the spread of a cassava mosaic disease pandemic. Plant Pathol..

[57] Polston JE, De Barro P, Boykin LM (2014). Transmission specificities of plant viruses with the newly identified species of the *Bemisia tabaci* species complex. Pest Manag. Sci..

[58] Ally HM (2019). What has changed in the outbreaking populations of the severe crop pest whitefly species in cassava in two decades?. Sci. Rep..

[59] Kalyebi, A. *et al.* Within-season changes in land use impact pest abundance in smallholder African cassava production systems. *Insects* (2021) **(Revised Submitted)**. 10.3390/insects12030269 PMC8005198 33810012

[60] Kalyebi A (2018). African cassava whitefly, *Bemisia tabaci*, cassava colonization preferences and control implications. PLoS ONE.

[61] Macfadyen S (2021). Landscape factors and how they influence whitefly pests in cassava fields across East Africa. Landsc. Ecol..

[62] Tay WT (2020). A high-throughput amplicon sequencing approach for population-wide species diversity and composition survey. bioRxiv.

[63] Manani DM, Ateka EM, Nyanjom SRG, Boykin LM (2017). Phylogenetic relationships among whiteflies in the *Bemisia tabaci*(Gennadius) species complex from major cassava growing areas in Kenya. Insects.

[64] Gottelli D, Marino J, Sillero-Zubiri C, Funk SM (2004). The effect of the last glacial age on speciation and population genetic structure of the endangered Ethiopian wolf (*Canis simensis*). Mol. Ecol..

[65] Sezonlin M (2006). Phylogeography and population genetics of the maize stalk borer *Busseola fusca* (Lepidoptera, Noctuidae) in sub-Saharan Africa. Mol. Ecol..

[66] Lehmann T (1999). The rift valley complex as a barrier to gene flow for *Anopheles gambiae* in Kenya. J. Hered..

[67] Schmidt H (2019). Transcontinental dispersal of *Anopheles gambiae* occurred from West African origin via serial founder events. Commun. Biol..

[68] Mairal M (2017). Geographic barriers and Pleistocene climate change shaped patterns of genetic variation in the Eastern Afromontane biodiversity hotspot. Sci. Rep..

[69] Freilich X (2016). Comparative Phylogeography of Ethiopian anurans: Impact of the Great Rift Valley and Pleistocene climate change. BMC Evol. Biol..

[70] Huhndorf MH, Peterhans JCK, Loew SS (2007). Comparative phylogeography of three endemic rodents from the Albertine Rift, east central Africa. Mol. Ecol..

[71] Matsubayashi KW, Ohshima I, Nosil P (2010). Ecological speciation in phytophagous insects. Entomol. Exp. Appl..

[72] Malka O (2018). Species-complex diversification and host-plant associations in *Bemisia tabaci*: A plant-defence, detoxification perspective revealed by RNA-Seq analyses. Mol. Ecol..

[73] Chen WB (2016). The draft genome of whitefly *Bemisia tabaci*MEAM1, a global crop pest, provides novel insights into virus transmission, host adaptation, and insecticide resistance. BMC Biol..

[74] Xie W (2018). The invasive MED/Q *Bemisia tabaci* genome: A tale of gene loss and gene gain. BMC Genomics.

[75] Katoh K, Standley DM (2013). MAFFT multiple sequence alignment software version 7: Improvements in performance and usability. Mol. Biol. Evol..

[76] Bolger AM, Lohse M, Usadel B (2014). Trimmomatic: A flexible trimmer for Illumina sequence data. Bioinformatics.

[77] Li H, Durbin R (2010). Fast and accurate long-read alignment with Burrows–Wheeler transform. Bioinformatics.

[78] Li H (2009). The sequence alignment/map format and SAMtools. Bioinformatics.

[79] Eaton DAR (2014). PyRAD: Assembly of de novo RADseq loci for phylogenetic analyses. Bioinformatics.

[80] Zheng XW (2012). A high-performance computing toolset for relatedness and principal component analysis of SNP data. Bioinformatics.

[81] Alexander DH, Novembre J, Lange K (2009). Fast model-based estimation of ancestry in unrelated individuals. Genome Res..

[82] Stamatakis A (2006). RAxML-VI-HPC: Maximum likelihood-based phylogenetic analyses with thousands of taxa and mixed models. Bioinformatics.

[83] Pickrell JK, Pritchard JK (2012). Inference of population splits and mixtures from genome-wide allele frequency data. PLoS Genet..

[84] Decker JE (2014). Worldwide patterns of ancestry, divergence, and admixture in domesticated cattle. PLoS Genet..

[85] Gompert Z (2014). Admixture and the organization of genetic diversity in a butterfly species complex revealed through common and rare genetic variants. Mol. Ecol..

