# Supplementary material for: On species delimitation, hybridization and population structure of cassava whitefly in Africa
Source: Sci Rep. 2021 Apr 12;11:7923. doi: 10.1038/s41598-021-87107-z (PMC8041820; doi:10.1038/s41598-021-87107-z)
Supplement: Supplementary file 1 — Supplementary Information 1. [file 41598_2021_87107_MOESM1_ESM.docx]

**On species delimitation, hybridization and population structure of cassava whitefly in Africa**

Elfekih S^1,a^, Tay WT^2a†^, Polaszek A^3^, Gordon KHJ^2^, Kunz D^4^, Macfadyen S^2^, Walsh TK^2^, Vyskočilová S^5^, Colvin J^5^, De Barro PJ^6^

1. CSIRO, Australian Centre for Disease Preparedness, Geelong, VIC, Australia
2. CSIRO, Black Mountain Laboratories, ACT, Australia
3. Department of Life Sciences, Natural History Museum, London, UK
4. The Gurdon Institute, University of Cambridge, Tennis Court Road, Cambridge CB2 1QN, UK
5. Natural Resources Institute, University of Greenwich, Kent, UK
6. CSIRO, Ecosciences Precinct, Brisbane, Australia

a. These authors contributed equally

**E-mail:** [Samia.elfekih@csiro.au](mailto:Samia.elfekih@csiro.au)

[Weetek.tay@csiro.au](mailto:Weetek.tay@csiro.au)

[A.Polaszek@nhm.ac.uk](mailto:A.Polaszek@nhm.ac.uk)

[Karl.gordon@csiro.au](mailto:Karl.gordon@csiro.au)

dk603@cam.ac.uk

[Sarina.Macfadyen@csiro.au](mailto:Sarina.Macfadyen@csiro.au)

[Tom.Walsh@csiro.au](mailto:Tom.Walsh@csiro.au)

[s.vyskocilova@gmail.com](mailto:s.vyskocilova@gmail.com)

[J.Colvin@greenwich.ac.uk](mailto:J.Colvin@greenwich.ac.uk)

[Paul.debarro@csiro.au](mailto:Paul.debarro@csiro.au)

**Correspondence:**  [weetek.tay@csiro.au](mailto:weetek.tay@csiro.au) **Tel:** +61-2-6246 4286

**Suppl. Fig. 1:** Sequence alignment summary of 14 SSA4-related sequences (8-21) from GenBank. SSA4 sequences of Wosula et al.^1^ are boxed in red. Sequences 1-4 are MEAM1 and MED partial mtCOI gene assembled from high-throughput sequencing (HTS) method^2,3^. SSA1 (sequence 5) and SSA2 (sequence 6) partial mtCOI gene as assembled from HTS method based on a single individual (see Kunz et al.^4^). SSA3 COI gene (sequence 7) as obtained from HTS of a single individual from University of Greenwich Natural Resources Institute laboratory stock (full data not shown). Wosula et al.^1^ reported SSA4 sequences were 8-10, and Berry et al.^5^ reported SSA4 sequences were 11-21. Red circles are indels. In three ‘putative SSA4’ sequences (8, 9, 10 of Wosula et al.^1^) and two SSA4 sequences (18 (Cam Ayos 2 WO3), 19 (Cam Ayos 1 WO2) of Berry et al.^5^), no indels were observed. Nine of the 11 Berry et al.^5^, SSA 4-equivalence sequences (labelled as ‘Sub-Saharan III’) showed indels. All SSA4 sequences of Wosula et al.^1^ and Berry et al. ^5^ shared ≥99% nucleotide identity. The reference SSA4 sequence used by Wosula et al.^1^ is sequence 11. Single nucleotide polymorphisms detected in sequence alignments were colour highlighted.


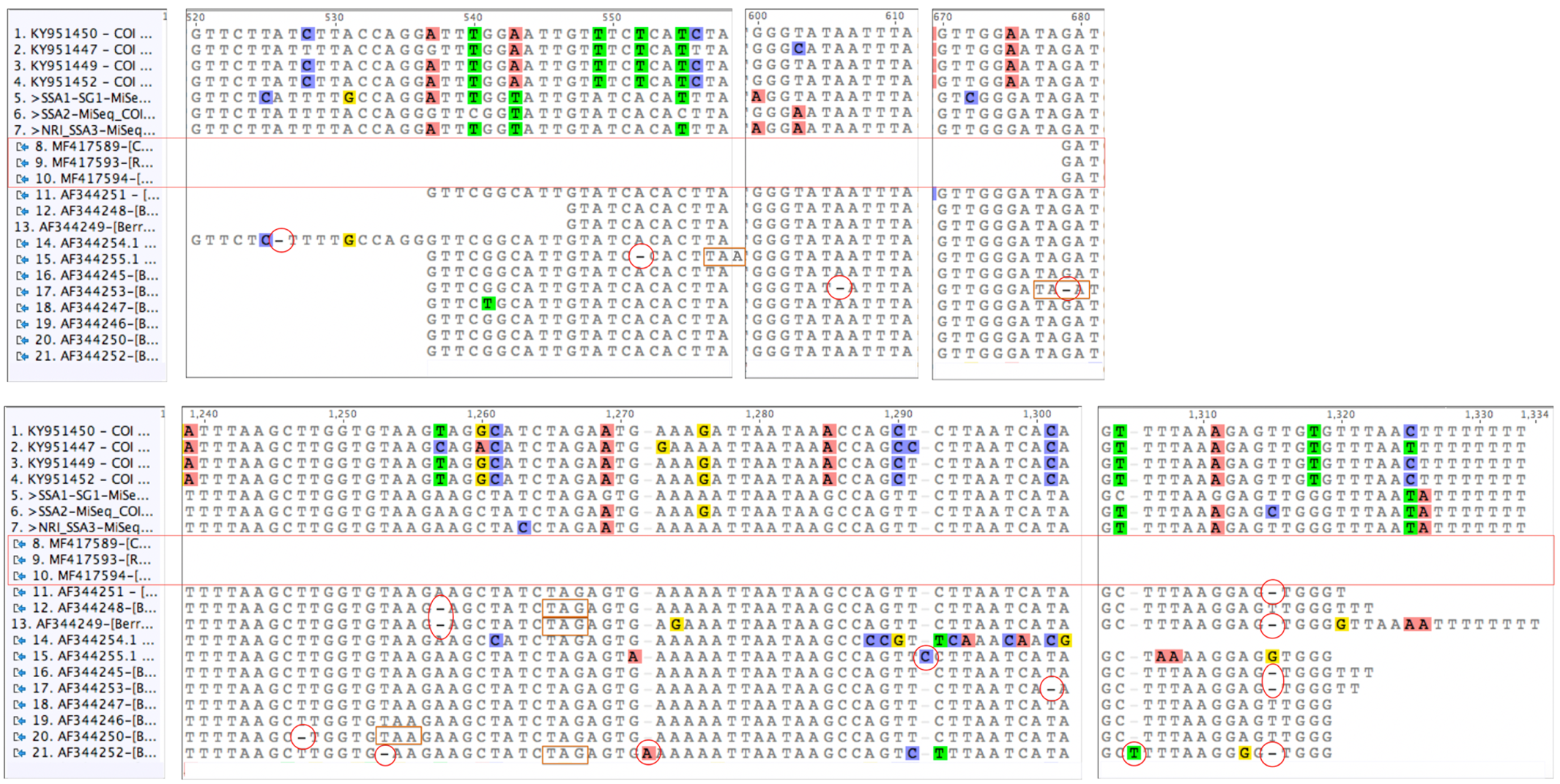


**Note:** Frameshift mutations leading to premature stop codons (‘TAA’ or ‘TAG’; indicated by rectangle boxes) in Berry et al.^5^ seqeunces are indicated. No premature stop codons were detected in the remaining five SSA4 sequences (i.e., AF344245, AF244246, AF244247, AF344251, AF344254) reported by Berry et al.^5^ despite presence of various INDELs (see main text).

**Suppl. Fig 2:** Schematic representation of partial mtCOI gene sequence alignments between sub-groups (SG) of the African cassava Bemisia whitefly SSA1 species. Numbers of nucleotide substitutions present within each alignment are indicated. Note that in: **[A]** three transition substitutions were observed between SSA1 individuals determined to belong to SG3 and SG5 (p-dist: 0.56%); **[B]** two tansition substitutions (p-dist: 0.37%) were observed between individuals in SG2; **[C]** three transitions and one transversion (p-dist: 0.75%)were observed between individuals that belonged to SG1; and **[D]** seven transition substitutions and one transversion substitution (p-dist: 1.5%) were observed between SG1 and SG2 individuals, for which mating experiments have failed to support these as being genetically distinct^6^.


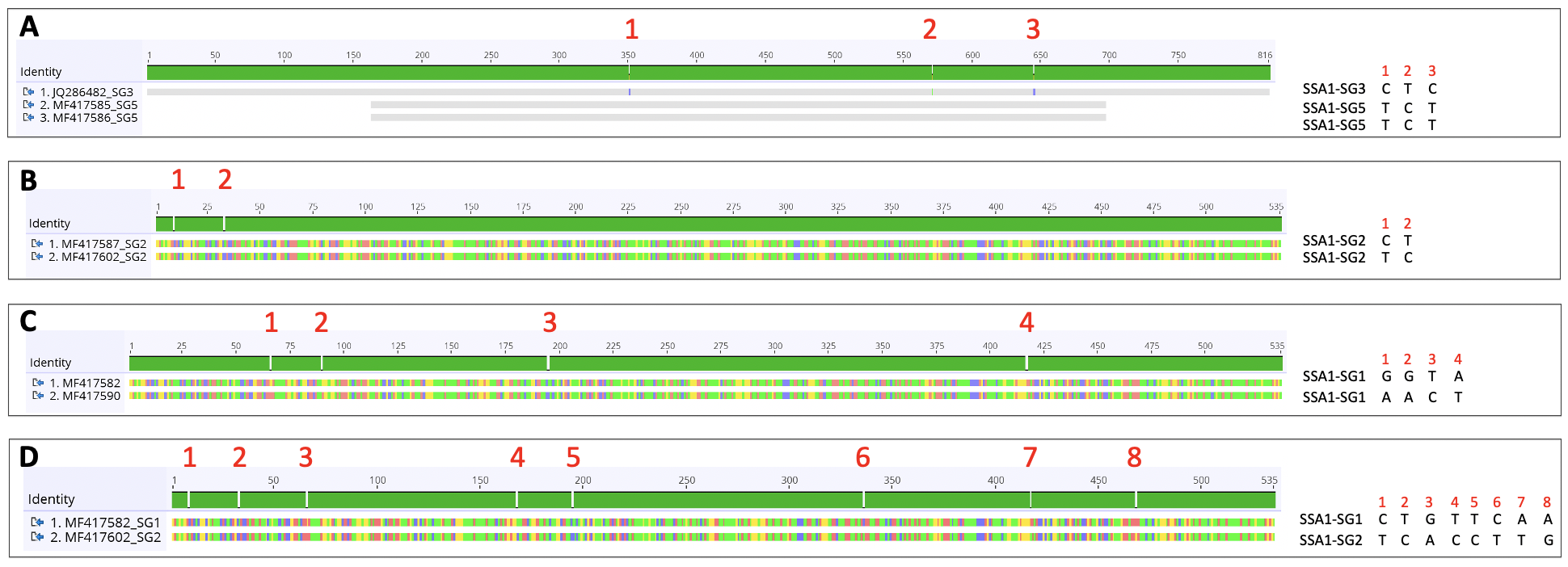


**Suppl. Fig 3:** Admixture plots (K= 2-4) and their respective cross-validation (CV) error estimates based on 14,358 geome-wide SNPs of 62 sub-Saharan African *Bemisia* cassava whitefly SSA1 and SSA2 cryptic species. CV error estimates indicated K=3 as the most optimal admixture plot, with the SSA2 and SSA1 indivdiuals maching their respective mtCOI halpotypes for species delimitation, and the SSA1 individuals showing NW (i.e., SSA-WA, SSA-ECA) and SE (i.e., SSA-CA. SSA-ESA) divide. Refere to the main text for detailed explanation.


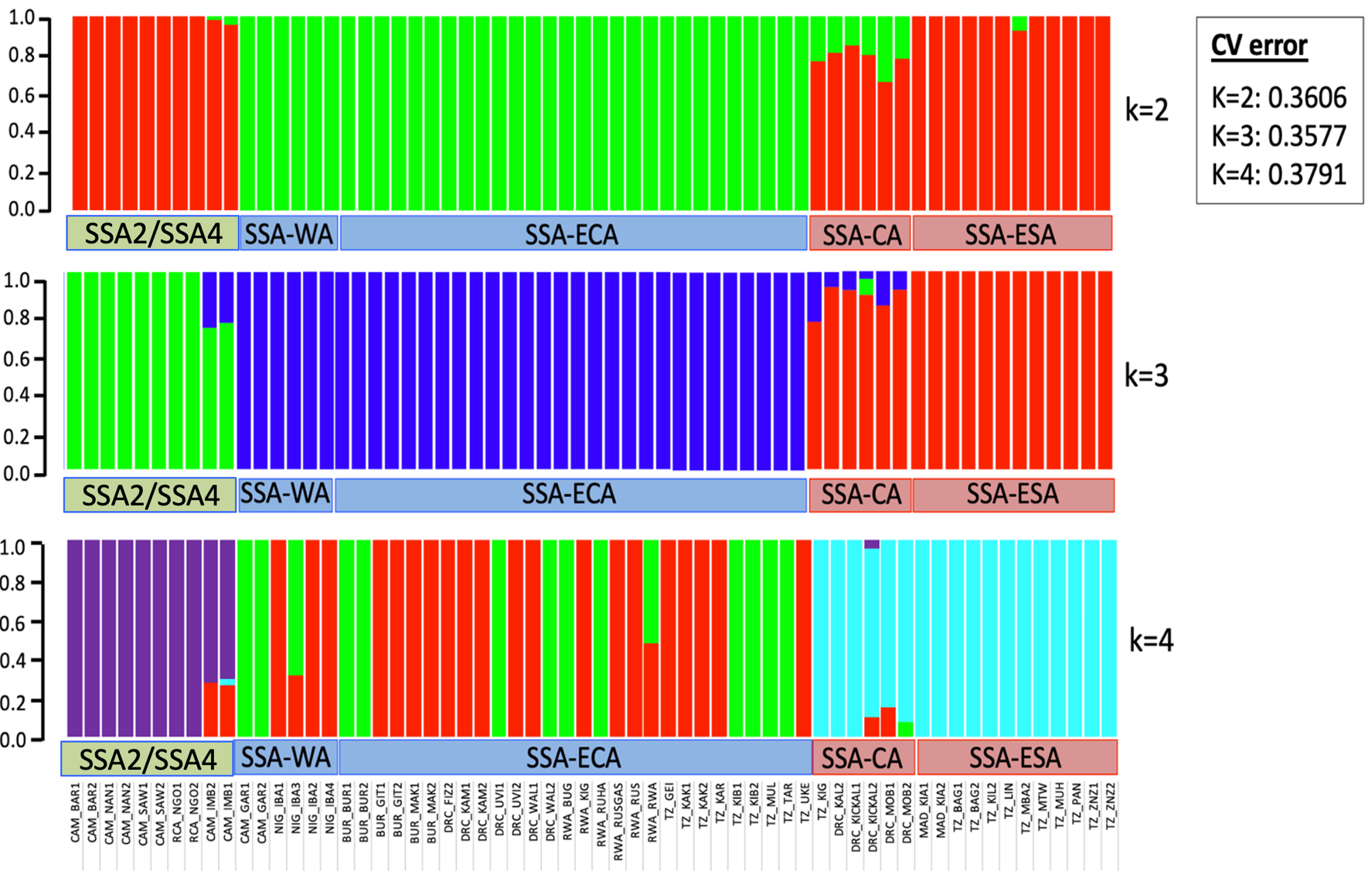


**Literature cited**

1. Wosula EN, *et a*l. Unravelling the Genetic Diversity among Cassava *Bemisia tabaci* Whiteflies Using NextRAD Sequencing. *Genome Biology and Evolution* 9: 2958-2973. doi: 10.1093/gbe/evx219 (2017)
2. Tay WT, Elfekih S, Court L, Gordon KH, De Barro PJ. Complete mitochondrial DNA genome of *Bemisia tabaci* cryptic pest species complex Asia I (Hemiptera: Aleyrodidae). *Mitochondrial DNA A DNA Mapp Seq Anal.* 27(2):972-973 (2016).
3. Tay WT, et al. Novel molecular approach to define pest species status and tritrophic interactions from historical *Bemisia* specimens. *Scientific Reports* 7: 429. Doi: 10.1038/s41598-017-00528-7 (2017).
4. Kunz D, *et al*. Take out the rubbish - Removing NUMTs and pseudogenes from the *Bemisia tabaci* cryptic species mtCOI database. *bioRxiv 724765; doi: https://doi.org/10.1101/724765* (2019).
5. Berry SD, et al. Molecular evidence for five distinct *Bemisia tabaci* (Homoptera : Aleyrodidae) geographic haplotypes associated with cassava plants in sub-Saharan Africa. *Annals of the Entomological Society of America* 97: 852-859. doi: Doi 10.1603/0013-8746(2004)097[0852:Meffdb]2.0.Co;2 (2004).
6. Mugerwa, H. *et al*. Whole-genome single nucleotide polymorphism and mating compatibility studies reveal the presence of distinct species in sub-Saharan Africa *Bemisia* *tabaci* whiteflies. *Insect Science*, doi:10.1111/1744-7917.12881 (2020).
